# ProClaT, a new bioinformatics tool for *in silico* protein reclassification: case study of DraB, a protein coded from the *draTGB* operon in *Azospirillum brasilense*

**DOI:** 10.1186/s12859-016-1338-5

**Published:** 2016-12-15

**Authors:** Elisa Terumi Rubel, Roberto Tadeu Raittz, Nilson Antonio da Rocha Coimbra, Michelly Alves Coutinho Gehlen, Fábio de Oliveira Pedrosa

**Affiliations:** 1 Laboratory of Bioinformatics, Professional and Technological Education Sector, Federal University of Paraná, Curitiba, PR Brazil; 2 Rua Dr. Alcides Vieira Arcoverde 1225, Curitiba, Paraná Brazil; 3 Department of Biochemistry and Molecular Biology, Federal University of Paraná, Curitiba, PR Brazil; 4 Av. Cel. Francisco H. dos Santos, s/n, Curitiba, Paraná Brazil

**Keywords:** Biological nitrogen fixation, Artificial neural networks, Protein classification, Nitrogenase, Nitrogenase associated NifO protein, *Azospirillum brasilense*, Operon *draTGB*

## Abstract

**Background:**

*Azopirillum brasilense* is a plant-growth promoting nitrogen-fixing bacteria that is used as bio-fertilizer in agriculture. Since nitrogen fixation has a high-energy demand, the reduction of N_2_ to NH_4_^+^ by nitrogenase occurs only under limiting conditions of NH_4_^+^ and O_2_. Moreover, the synthesis and activity of nitrogenase is highly regulated to prevent energy waste. In *A. brasilense* nitrogenase activity is regulated by the products of *draG* and *draT*. The product of the *draB* gene, located downstream in the *draTGB* operon, may be involved in the regulation of nitrogenase activity by an, as yet, unknown mechanism.

**Results:**

A deep *in silico* analysis of the product of *draB* was undertaken aiming at suggesting its possible function and involvement with DraT and DraG in the regulation of nitrogenase activity in *A. brasilense*. In this work, we present a new artificial intelligence strategy for protein classification, named ProClaT. The features used by the pattern recognition model were derived from the primary structure of the DraB homologous proteins, calculated by a ProClaT internal algorithm. ProClaT was applied to this case study and the results revealed that the *A. brasilense draB* gene codes for a protein highly similar to the nitrogenase associated NifO protein of *Azotobacter vinelandii*.

**Conclusions:**

This tool allowed the reclassification of DraB/NifO homologous proteins, hypothetical, conserved hypothetical and those annotated as putative arsenate reductase, ArsC, as NifO-like. An analysis of co-occurrence of *draB*, *draT*, *draG* and of other *nif* genes was performed, suggesting the involvement of *draB* (*nifO*) in nitrogen fixation, however, without the definition of a specific function.

**Electronic supplementary material:**

The online version of this article (doi:10.1186/s12859-016-1338-5) contains supplementary material, which is available to authorized users.

## Background

*Azospirillum brasilense* is a diazotrophic organism used as commercial inoculants, since it promotes plant growth [1]. As a nitrogen-fixing bacterium, *A. brasilense* has a specific metabolic pathway for the conversion of gaseous dinitrogen into ammonia. The N_2_ is fixed under limiting conditions of NH_4_^+^ and O_2_, through the activity of nitrogenase [2]. A post-translational control of nitrogenase occurs via the DraG-DraT system, in which the DraT enzyme (dinitrogenase reductase ADP-ribosyltransferase) acts in the nitrogenase shutdown by inactivating the NifH (dinitrogenase reductase) in response to the presence of ammonium ions in the environment, while the DraG enzyme (dinitrogenase reductase activating-glycohydrolase) restores the activity of NifH, after ammonium ions consumption [3, 4]. The DraT and DraG enzymes are encoded by the *draTG* genes, of the *draTGB* operon in *A. brasilense* [5]*.* The *draB* gene was annotated as coding a putative arsenate reductase [5] [GenBank: CCC97498]. However, this function for the *draB* gene product of *Azospirillum brasilense* has never been confirmed to date. There is evidence that a homologous protein in *Rhodospirillum rubrum* seems to regulate the activity of DraG [6]. The *draB* gene is homologous to *nifO* of *A. vinelandii* and *arsC* of *E. coli* [7]. The *A. vinelandii* nitrogenase-associated NifO protein, part of operon *nifBfdxNnifOQ*, has a role in regulating the activity of nitrate reductase, whereas mutants NifO^−^ cannot fix nitrogen in the presence of low concentrations of nitrate [8, 9].

To test the hypothesis that the *draB* gene codes for a NifO-like protein, since DraB protein has no known homologous in the Gene Ontology database, we developed a strategy named ProClaT - Protein Classifier Tool - for the reclassification of DraB/NifO homologous proteins, hypothetical, conserved hypothetical and those annotated as putative arsenate reductase, ArsC, as NifO-like.

A supervised pattern recognition approach was developed with a neural network as classifier. Also, the relationship and co-occurrence of *draB* with other genes related to nitrogen fixation, the minimum *nif* gene set, *nifHDKENB* [10], and with the *draT* and *draG* genes involved in the control of nitrogenase activity was determined by the Pearson Correlation Analysis.

## Methods

ProClaT is a new machine learning approach to classify proteins based on protein sequence features and conserved domains. ProClaT was used to classify *draB* gene products and to discover NifO-like proteins.

### Data

ProClaT was applied to 2,773 complete bacterial genomes obtained from the NCBI database [11] via FTP, containing 5,182 GenBank data downloaded in July 2014. The download file size was 78.1 GB.

### ProClaT pattern recognition sequence-based features

The features used by the pattern recognition model are divided into three categories:

1. **Amino acid composition**

The relative occurrence of each amino acid residue and its number in each functional group (polar positively charged, polar negatively charged, nonpolar and hydrophobic) was calculated by dividing the number of occurrence of each amino acid residue by the total number of amino acid residues in the protein. The protein sequence length was also used to compose its features.

2) **Consensus region alignment scores**

The protein consensus region was used for determining the alignment score of each protein sequence. A self-alignment function and the global and local alignment sequence scores, determined by the Needleman-Wunsch algorithm (identity and positive scores), were used as additional features.

3) **Protein physico-chemical properties**

The protein physicochemical features used to develop ProClaT were the isoelectric point (pI), charge, nominal mass, aromaticity, instability, hydropathy, entropy and energy.

Isoelectric point: The estimated pI for an amino acid sequence was calculated with Matlab and the Bioinformatics Toolbox™, using the pK values described on http://www.mathworks.com/help/bioinfo/ref/isoelectric.html.

Charge: The estimated charge of a protein in a given pH was calculated by the same Matlab function of the Bioinformatics Toolbox™ as for the pI described above. The default value was taken as the typical intracellular pH of 7.2.

Nominal mass: The expected protein nominal mass was also calculated by a Matlab function of the Bioinformatics Toolbox™, which analyzes a peptide sequence (http://www.mathworks.com/help/bioinfo/ref/isotopicdist.html).

Aromaticity: The aromaticity value of a protein was calculated according to Lobry [12], and consider the relative frequency of Phe + Trp + Tyr.

Instability: The protein instability index was calculated according to Guruprasad et al. [13]. In this procedure a value above 40 means that the protein is unstable or has a short half-life.

Hydropathy or GRAVY (Grand Average of Hydropathy) Index: The protein GRAVY index was calculated according to the Kyte and Doolittle methodology [14]. This index reveals the solubility of a protein, where a positive GRAVY value corresponds to a hydrophobic protein and a negative GRAVY value corresponds to a hydrophilic protein. The GRAVY value of a peptide/protein is calculated by adding the values of hydropathy of each amino acid, divided by the total number of residues of the sequence.

Entropy and Energy: In this context, the descriptors Energy and Entropy represent, respectively, the degree of uniformity and disorder of each protein sequences. Co-occurrence matrices 3 × 3 were generated from amino acids based on the sequence, and for each entry, the sequence was read from the right to the left and stored in a 3 × 3 amino acids arrangement. Based on this list, the combinations in pairs were analyzed one by one, and in case of co-occurrence, the count and recording of data was updated. This calculation was based on the Haralick methodology [15] called “matrix of co-occurrence”, developed for the description of textures images based on second-order statistics.

The Aromaticity, Instability and Hydropathy were calculated using the package Biopython. The features extraction is part of the tool. Table 1 shows the summary of the three feature categories, including the number of features generated and the functions used to extract them.

**Table 1:** Features of the ProClaT pattern recognition model

Feature category	Number of features	Function (Matlab or Python)
AA composition^a^		
AA composition^a^	20	aacount (sequence)
AA functional property^a^	5	codon2aa (sequence)
Protein length	1	length (sequence)
Scores alignment with consensus region		
Self align with consensus region	1	selfalign (sequence,CSeq)
Global alignment score with consensus region	2	getIdentity (sequence,CSeq,‘G’)
Local alignment score with consensus region	2	getIdentity (sequence,CSeq,‘L’)
Protein physico-chemical properties		
pI	1	isoelectric (sequence) (first returned value)
Charge	1	isoelectric (sequence) (second returned value)
Nominal mass	1	isotopicdist (sequence)
Aromaticity	1	ProtParam.ProteinAnalysis (seq).aromaticity() (python)
Instability	1	ProtParam.ProteinAnalysis (seq).instability_index() (python)
Hydropathy	1	ProtParam.ProteinAnalysis (seq).gravy() (python)
Entropy	1	function developed in python
Energy	1	function developed in python

^a^ *AA* amino acid

### ProClaT algorithm

**Fig. 1:**
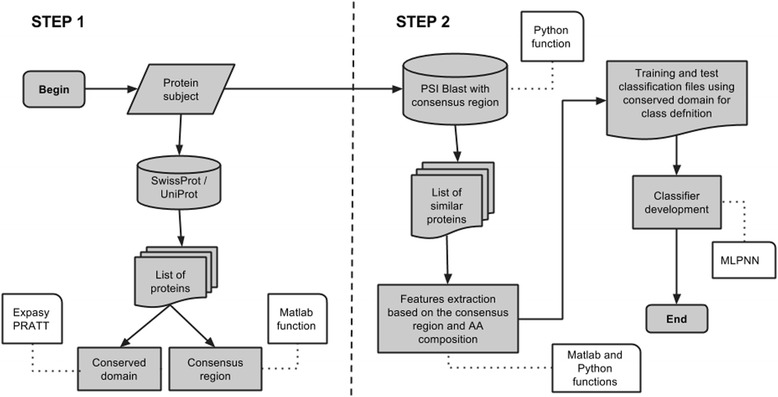
Flowchart representing the algorithm to develop ProClaT. In the first step, the domain conserved protein and the consensus region are generated. In the second step, a search is performed in NCBI NR with the generated region consensus as query. With the list of similar proteins, the features are extracted and the classifier is trained and tested

ProClaT development algorithm flow can be seen in Fig. 1.

**Fig. 2:**
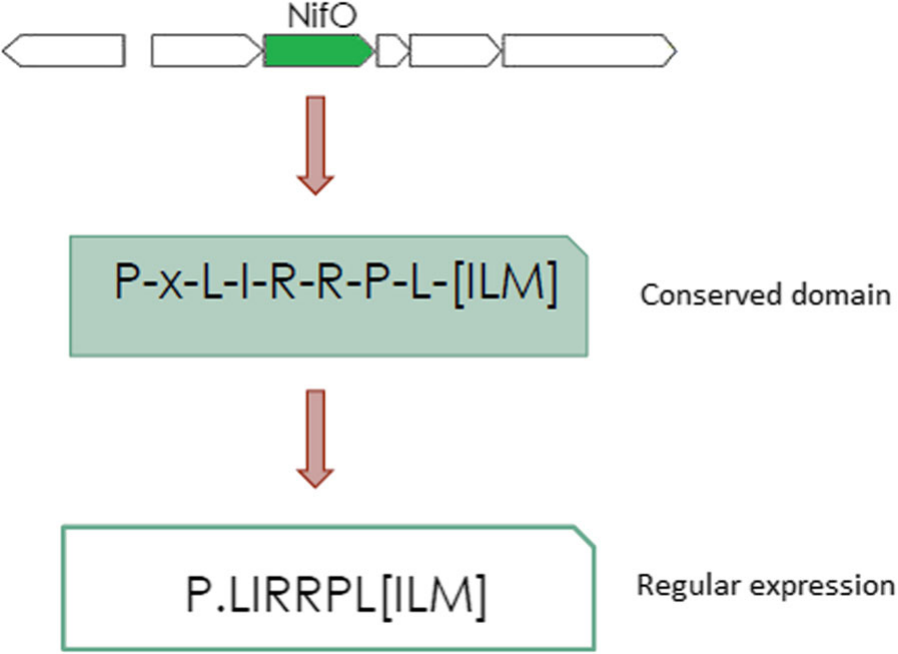
Conserved domain of NifO-like proteins and the corresponding *regular expression*. The conserved domain was generated with the Expasy PRATT tool after the refinement phase

The protein conserved domain and consensus region were determined using the curated sequences protein deposited in the SwissProt database. Since there are no reviewed NifO proteins in the SwissProt database, the NifO proteins deposited in the Uniprot database were used. To generate the conserved domain of a protein, we used the Expasy PRATT tool [16]. This conserved domain may be a common ancestor consequence with the evolutionary pressure to maintain important residue in the active site and other relatively important parts of the protein and are useful to identify new family members [16]. The conserved NifO domain generated by PRATT defined a *regular expression* (Fig. 2). Considering that the number of coded amino acids residues in proteins is 20, the probability of random occurrence of this amino acid sequence is 1.1719*10^−10^.

**Fig. 3:**
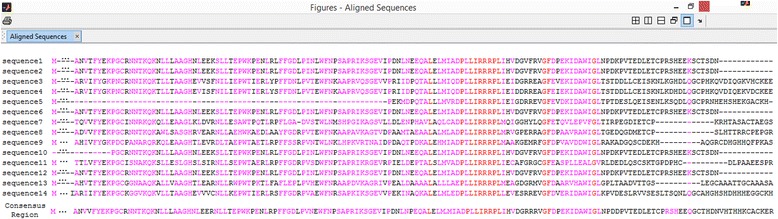
NifO-like consensus region. Generated from the multiple alignment of the NifO proteins

The consensus region (Fig. 3) was used as a query in a PSI-Blast search in the NR NCBI protein library, returning 5,000 hits of similar proteins using the Blast default values. The *regular expression* allowed the identification of proteins among the 5,000 that have the conserved domain. These proteins were submitted to the feature extractor and were used to create the classifier training and test files, as the Label 1 class (“TRUE to NifO”). To compose the Label 0 class (“FALSE to NifO”), were used the proteins with the lowest similarity levels that do not have the conserved domain.

ProClaT was parameterized in order to classify the NifHDK, NifENB, DraT and DraG proteins. Instead of a single TRUE/FALSE classifier, its returns 1 for NifH, 2 for NifD, 3 for NifK, 4 for NifE, 5 for NifN and 6 for NifB. For DraT and DraG, it returns 1 and 2 respectively.

ProClaT only ranks candidate proteins, with at least 0.2 of identity calculated by a self-alignment function. This function returns the average of the global alignment of two sequences using the Needleman-Wunsch algorithm:

1 $$ selfalign=\frac{\frac{globalAlign\left(seq1,seq2\right)}{globalAlign\left(seq1,seq1\right)}+\frac{globalAlign\left(seq1,seq2\right)}{globalAlign\left(seq2,seq2\right)}}{2} $$

### Implementation

As shown in Table 2, ProClaT was developed in the programming language Matlab ®, which also worked as Integrated Development Environment (IDE), using the Bioinformatics Toolbox™. Some feature extractions were performed in Python using the Biopython package [17].

**Table 2:** Software versions

Software	Version	Application
Matlab	r2012B (8.0.0.783)	Functions to get the conserved domain, features extraction and create the classifier.
Python	3.4.2	Functions to perform PSI-Blast and features extraction.
Expasy PRATT	2.1	Generate the protein conserved domains.
Weka	3.6.12	Test of the classifiers algorithms.

The ProClaT algorithm for supervised classification chosen was the Multilayer Perceptron Neural Network (MLPNN), a feed-forward back-propagation machine learning method [18]. MLPNN returned the best results, according to the Weka data mining software [19], as shown in Table 3. In this case, the implementation without the cross-validation technique showed better results. For the algorithm selection, were considered the best algorithms according to the Top 10 data mining algorithms identified by the IEEE International Conference on Data Mining (ICDM) presented in December 2006 in Hong Kong [20].

**Table 3:** Correctly classified proteins by Weka algorithms

Algorithm	Options	Correctly classified instances without cross-validation	Correctly classified instances with cross-validation
Multilayer Perceptron	-L 0.3 –M 0.2 –N 500 –V 0 –S 0 –E 20 –H a	99.61 %	99.41 %
Simple Cart	-S 1 –M 2.0 –N 5 –C 1.0	99.09 %	99.22 %
Nnge	-G 5 –I 5	99.09 %	99.02 %
J48	-C 0.25 –M 2	98.96 %	98.71 %
Ada BoostM1	-P 100 –S 1 –I 0 –W weka.classifiers.trees. DecisionStump	32.51 %	33.35 %
Naive Bayes	-	99.22 %	98.90 %

Using the default parameters proposed by Weka, the neural network training and test files were submitted to the six algorithms above. MLPNN showed the best number of correctly classified proteins

For the *nifO* neighborhood analysis, we identified the *nifO* neighboring genes in a five window genes upstream and downstream using ProClaT.

## Results and discussion

**Fig. 4:**
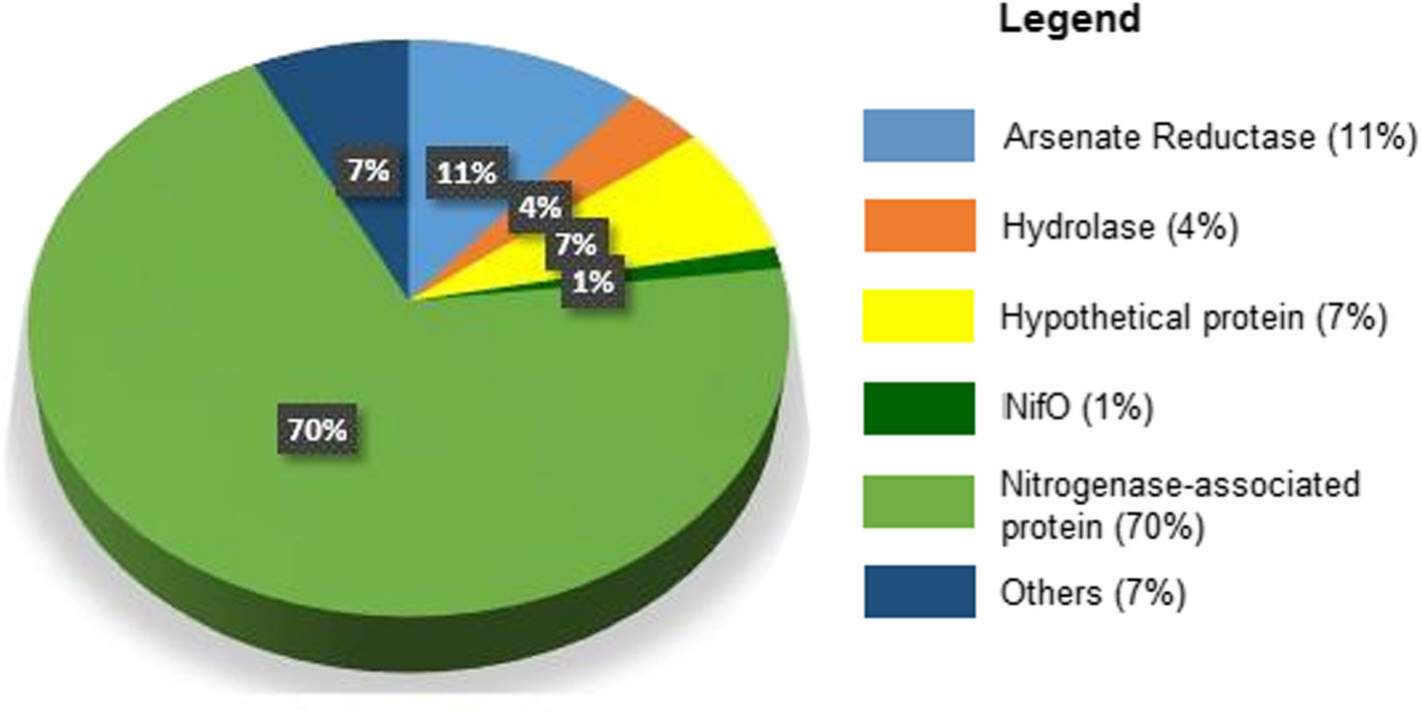
Original annotation of all complete genome NifO-like proteins. Of the 82 proteins classified as NifO-like with ProClaT, most correspond to nitrogenase-associated protein. The proteins annotated as arsenate reductase, hypothetical and others, totaling 21 proteins, might be re-classified as NifO-like, also

ProClaT was applied to analyze 2,773 complete bacterial genomes and found 82 NifO-like proteins belonging to 76 genomes, representing 56 bacterial species, including the DraB protein of *Azospirillum brasilense*. The original annotation of these proteins is shown in Fig. 4, and the reclassification by ProClaT of these proteins is shown in Additional file 1.

The product of the *PST1305* gene of *Pseudomonas stutzeri* A1501, classified as NifO-like with ProClaT, was suggested to participate in biological nitrogen fixation, probably involved in electron transport or in an oxygen protection mechanism for nitrogenase [21]. The authors considered this gene product to be required for optimal nitrogenase activity of *Pseudomonas stutzeri* A1501.

Moreover, the *A. vinelandii* NifO protein was also classified as NifO-like, as expected. Laboratory tests suggests that this protein has a role on ammonium repression of the nitrite-nitrate (*nasAB*) assimilatory operon of *Azotobacter vinelandii* [9].

Considering that the *nifO* gene is involved in the molybdenum (Mo) metabolism in *A. vinelandii*, and that nitrogenase and nitrate reductase contain Mo cofactors, NifO may be involved in regulating the distribution of Mo towards the synthesis of nitrogenase FeMoco or the synthesis of the nitrate reductase cofactor [9].

ProClaT was applied also in the classification of NifHDK, NifENB, DraT and DraG in order to confirm its general applicability.

The Additional file 2 lists all bacterial species containing at least five essential *nif* genes, and the presence of *nifHDK*, *nifENB*, *nifO*, *draT* and *draG* genes, according to ProClaT. Of the 80 bacterial species (or 119 strains) that have the six essential *nif* genes, 42 (or 61 strains) or 50 % co-occur with *nifO,* including *Acidithiobacillus ferrivorans*, *Bradyrhizobium japonicum*, *Burkholderia xenovorans*, *Magnetospirillum magneticum*, *Pseudomonas stutzeri* and *Rhodospirillum rubrum. H*owever, 41 bacterial species (or 58 strains) have no *nifO*-like genes, including *Herbaspirillum seropedicae*, *Klebsiella oxytoca, Enterobacter sp* and *Burkholderia phenoliruptrix*.

**Fig. 5:**
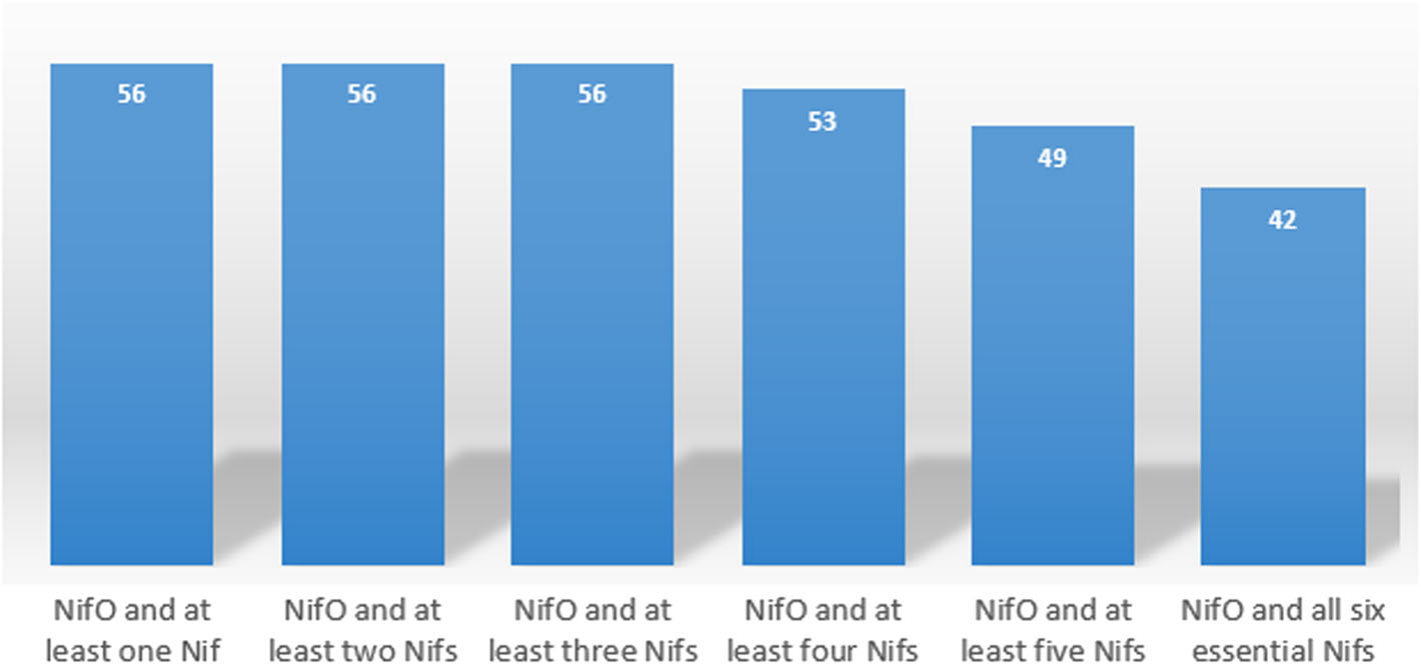
Bacterial species containing gene coding for NifO-like and for Nif proteins. ProClaT identified 56 bacterial species containing genes coding for nifO-like. All belong to a genome containing at least three genes coding for an essential Nif protein. Fifty-three species contain at least 4 *nif* genes, 49 contain at least 5 *nif* genes and 42 contain all the six essential *nif* genes

All genes coding for NifO-like proteins identified by ProClaT belong to bacteria having at least three of the essential *nif* genes. Figure 5 shows the number of bacterial species containing genes coding for NifO-like proteins associated with genes coding for essential Nif proteins in the complete genomes analyzed.

**Fig. 6:**
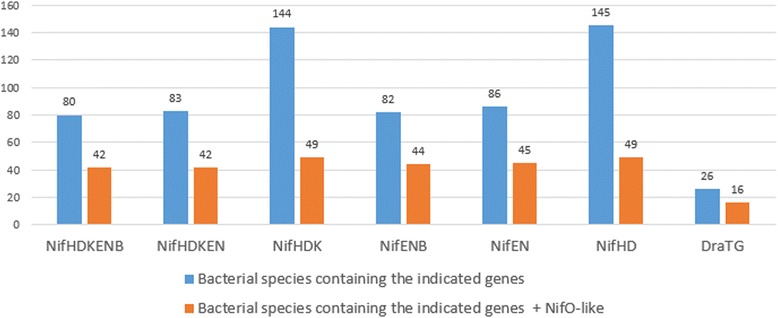
Bacterial species containing gene groups with the presence of *nifO*. In blue, the number of species of bacterial complete genomes containing the genes indicated below, and in red, the number of the species containing these genes in addition with the gene coding for NifO- like

Figure 6 shows the number of gene groups found in the complete genome with ProClaT, analyzing the bacterial species.

Interestingly, the species *Azospirillum brasilense*, *Azospirillum lipoferum* and *Azotobacter vinelandii* have two genes coding for NifO-like protein, according to ProClaT. Worth noting that no genes coding for NifO-like proteins were found in plasmids.

**Fig. 7:**
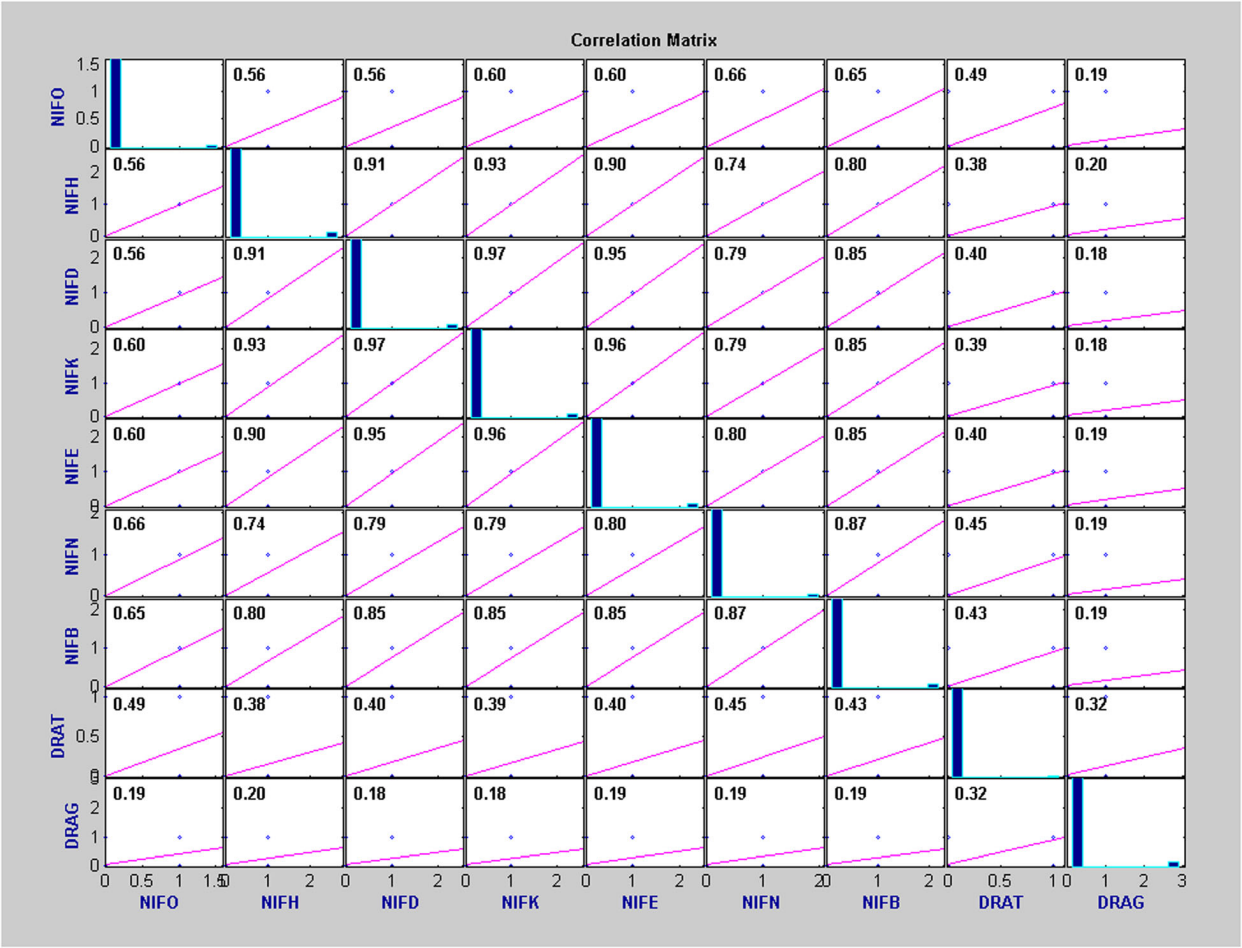
Pearson Correlation Coefficient of the genes co-occurrence in complete bacterial genomes. The *nifO*, *nifH*, *nifD*, *nifK*, *nifE*, *nifN*, *nifB*, *draT* and *draG* genes were analyzed. The Pearson Correlation Coefficient is a well-established measure of correlation with range from +1 (perfect correlation) to −1 (perfect but negative correlation), in which 0 is the absence of a relationship [29]. The highest *p -value* found was 6.7*10^−39^, indicating that all pairs of variables have correlation significantly different from zero. Image generated by Matlab

The co-occurrence of the genes coding for NifO-like, NifHDK-like, NifENB-like, DraT-like and DraG-like proteins was determined using the Pearson Correlation Coefficient. Figure 7 shows this correlation for the complete bacterial genomes analyzed.

The co-occurrence correlation of *nifO* and other *nif* genes is higher than that observed with the *draT* and *draG* genes.

The Pearson Correlation Coefficient of *nifO* co-occurrence with all the six *nif* genes is 0.6350, and with the presence of both *draT* and *draG* genes is 0.4544.

The analysis of neighborhood genes, in a five window genes upstream and downstream, showed that *nifO* is regularly located close to at least one *nif* gene, as well as to *draT* or *draG* genes. Table 4 shows the number of the *nif* genes present in the *nifO* neighborhood.

**Table 4:** Genes present in the *nifO* neighborhood

Gene	Absolute number of occurrences of the genes in the *nifO* neighborhood
*nifH*	24
*nifD*	12
*nifK*	8
*nifE*	1
*nifN*	0

The number of genes present in the *nifO* neighborhood, in a five window gene upstream and downstream

### ProClaT comparison and validation

Table 5 compares the NifO-like proteins predicted by ProClaT with those predicted by cut-off score, conserved domain and both cut-off score and conserved domain.

**Table 5:** Sensitivity and specificity of protein prediction methods

Method	TP^a^	TN^b^	FP^c^	FN^d^	Calculated sensitivity (%)	Calculated specificity (%)
1. Cut-off score (>30 % local identity and > 50 % positive)	229	2704	0	67	77.36	100
2. Conserved domain	231	2704	10	55	80.77	99.63
3. Conserved domain with cutoff score	219	2704	0	77	73.99	100
4. ProClaT	289	2704	0	7	97.64	100

^a^ *TP* true positive

^b^ *TN* true negative

^c^ *FP* false positive

^d^ *FN* false negative

A PSI-Blast was performed on the NCBI NR protein library, using the consensus region of NifO as input query. It returned 3,000 hits of similar proteins, which 296 are NifO-like, after curation. All these proteins were submitted to the above methods. ProClaT showed the best sensitivity.

ProClaT was applied to all NifHDKENB proteins deposited in the SwissProt database to determine its accuracy in identifying homologous proteins (Table 6).

**Table 6:** NifHDKENB proteins identification by ProClaT

Protein	Class	Number of curated proteins	ProClaT Hits	Accuracy
NifH	1	92	91	98.91 %
NifD	2	23	22	95.65 %
NifK	3	17	16	94.12 %
NifE	4	14	14	100 %
NifN	5	10	10	100 %
NifB	6	13	12	92.31 %

A search was performed in the SwissProt protein database by the proteins name NifHDK and NifENB, curated manually. Each found protein was applied to ProClaT, and the accuracy was calculated. The average of success rate was 96.83 %

Although of high accuracy, ProClaT specificity can be improved. The observed average low error rate (3.17 %) was probably due to the fact that a small number of curated NifHDKENB proteins was available in biological databases to train the ProClaT neural network.

### DraB classification with published protein prediction tools

Since *A. brasilense* DraB protein has no homologous in the GO database, as revealed by BLAST performed with the AmiGO web tool [22], the functional classification services based on GO terms were not specific. The ConFunc tool [23] predicted for the DraB protein the following terms: 1) GO: 0008794 (ontology: molecular function, description: arsenate reductase glutaredoxin activity) with probability of 0.667 and 2) GO: 0006351 (ontology: biological process, description: transcription, DNA- template) with probability 0.306. With the Blast2GO tool [24], the terms suggested to the DraB protein were: 1) GO: 0055114 (ontology: biological process, description: oxidation-reduction process) and 2) GO: 0016491 (ontology: molecular function, description: oxidoreductase activity). Other Bioinformatics tools suggest that DraB can belong to the families arsenate reductase-like (InterPro [25] and PANTHER [26]), thioredoxin-like fold (InterPro [25], Pfam [27] and PROSITE [28]) or to the family annotated, but not proven, as nitrogenase-associated protein (InterPro [25]). The protein prediction methods based on its tertiary structure are not recommended in this case, since there are no models of tertiary structure of DraB/NifO homologous obtained via experiments laboratory in protein structure databases.

## Conclusions

A new efficient tool for protein classification - ProClaT - is described and tested. In this *in silico* study, ProClaT revealed that the *draB* gene of *Azospirillum brasilense* codes for a NifO-like protein. There is evidence that *A. vinelandii* NifO is possibly involved in regulating the distribution of Mo towards the synthesis of nitrogenase FeMoco or the synthesis of the nitrate reductase cofactor [9].

All the genes coding for NifO-like found with ProClaT belong to bacteria having at least three of the six essential *nif* genes, *nifHDK* and *nifENB* [10]. With the correlation analysis of co-occurrence of these genes in complete bacterial genomes, we observed that the *nifO/draB* gene has a higher correlation coefficient with the essential *nif* genes than with *draT* and *draG*, whose products is involved in controlling nitrogenase activity in response to ammonium levels.

Analysis of the neighborhood revealed that *nifO* may have both *nif* and/or *draT* and *draG* genes as neighbors, but no clear pattern was identified.

Of the 80 bacterial species analyzed containing the six essential *nif* genes, 42 also contain the *nifO* gene. However, 41 diazotrophic bacterial species have no *nifO-like* genes, which suggests that *nifO* is not essential for the nitrogen fixation by nitrogenase.

ProClaT found nine genes annotated as arsenate reductase, six as hypotheticals and six with variable names in complete bacterial genomes. This suggests that these gene products should be reclassified as NifO-like.

ProClaT was developed to reclassify the DraB protein *vis a vis* the NifO-like proteins and to approach its biological functions.

ProClaT was tested with curated Nif proteins and showed average hit rate of 96.83 % in classifying known Nif proteins, confirming that it can be useful in the (re)classification of other proteins. Thus, ProClaT has a much wider application as revealed by its validation with the defined essential nitrogen fixation proteins.

## Additional files

Additional file 1: Original annotation of reclassified proteins as NifO-like by ProClaT. The following list shows how the proteins classified in NifO-like are currently annotated, analyzing complete bacterial genomes. It is worth noting that less than 2 % of the genes were originally annotated as *nifO*. (XLSX 12 kb)
Additional file 2: List of bacterial species having at least 5 genes *nif* and the presence of the genes *nif*, *nifO*, *draT* and *draG.* In the list below are all bacterial species that contain at least five essential *nif* genes according to ProClaT, analyzing the complete genomes of bacteria. The columns indicate the presence of *nifHDK*, *nifENB*, *nifO*, *draT* and *draG* genes. (XLSX 14 kb)

## References

[1] Hungria M, Campo RJ, Souza EM, Pedrosa FO (2010). Inoculation with selected strains of *Azospirillum brasilense* and *A. lipoferum* improves yields of maize and wheat in Brazil. Plant Soil.

[2] Postgate JF (1982). The fundamentals of nitrogen fixation.

[3] Zumft WG, Castillo F (1978). Regulatory properties of the nitrogenase from *Rhodopseudomonas palustris*. Arch Microbiol.

[4] Huergo LF, Pedrosa FO, Muller-Santos M, Chubatsu LS, Monteiro RA, Merrick M, Souza EM (2012). PII signal transduction proteins: pivotal players in post-translational control of nitrogenase activity. Microbiology.

[5] Zhang Y, Burris RH, Roberts GP (1992). Cloning, sequencing, mutagenesis, and functional characterization of *draT* and *draG* genes from *Azospirillum brasilense*. J Bacteriol.

[6] Liang J, Nielsen GM, Lies DP, Burris RH, Roberts GP, Ludden PW (1991). Mutations in the *draT* and *draG* Genes of *Rhodospirillum rubrum* result in loss of regulation of nitrogenase by reversible ADP-Ribosylation. J Bacteriol.

[7] Zhang Y, Pohlmann EL, Halbleib CM, Ludden PW, Roberts GP (2001). Effect of PII and Its Homolog GlnK on Reversible ADP-Ribosylation of Dinitrogenase Reductase by Heterologous Expression of the *Rhodospirillum rubrum* dinitrogenase reductase ADP-ribosyl transferase-dinitrogenase reductase-activating glycohydrolase regulatory system in *Klebsiella pneumonia*. J Bacteriol.

[8] Quiñones FR, Bosh R, Imperial J (1993). Expression of the *nifBfdxNnifOQ* Region of *Azotobacter vinelandii* and Its Role in Nitrogenase Activity. J Bacteriol.

[9] Gutierrez JC, Santero E, Tortolero M (1997). Ammonium repression of the nitrite-nitrate (*nasAB*) assimilatory operon of *Azotobacter vinelandii* is enhanced in mutants expressing the *nifO* gene at high levels. Mol Gen Genet.

[10] Dos Santos PC, Fang Z, Mason SW, Setubal JC, Dixon R (2012). Distribution of nitrogen fixation and nitrogenase-like sequences amongst microbial genomes. BMC Genomics.

[11] NCBI GenBank FTP. ftp://ftp.ncbi.nlm.nih.gov/genbank/genomes/ (2015). Accessed 19 Apr 2015.

[12] Lobry JR, Gautier C (1994). Hydrophobicity, expressivity and aromaticity are the major trends of amino-acid usage in 999 *Escherichia coli* chromosome-encoded genes. Nucleic Acids Res.

[13] Guruprasad K, Reddy BV, Pandit MW (1990). Correlation between stability of a protein and its dipeptide composition: a novel approach for predicting in vivo stability of a protein from its primary sequence. Protein Eng.

[14] Kyte J, Doolittle RF (1982). A simple method for displaying the hydropathic character of a protein. J Mol Biol.

[15] Haralick RM (1979). Statistical and structural approaches to texture. Proc IEEE.

[16] Jonassen I, Collins JF, Higgins DG (1995). Finding flexible patterns in unaligned protein sequences. Protein Sci.

[17] Cock PJA, Antao T, Chang JT, Chapman BA, Cox CJ, Dalke A, Friedberg I, Hamelryck T, Kauff F, Wilczynski B, de Hoon MJL (2009). Biopython: freely available python tools for computational molecular biology and bioinformatics. Bioinformatics.

[18] Jain AK, Duin RPW, Mao J (2000). Statistical pattern recognition: a review. IEEE Trans Pattern Anal Mach Intell.

[19] Hall M, Frank E, Holmes G, Pfahringer B, Reutemann P, Witten I (2009). The WEKA data mining software: an update. ACM SIGKDD Explorations News.

[20] Wu X, Kumar V, Quinlan JR, Ghosh J, Motoda QYH, Mclachlan GJ, Ng A, Liu B, Yu PS, Zhou Z, Steinbach M, Hand DJ, Steinberg D (2008). Top 10 algorithms in data mining. Knowl Inf Syst.

[21] Fan H, Yan Y, Li Y, Ping S, Zhang W, Chen M, Lin M, Lu W (2009). Analysis of a new nitrogen fixation gene in *Pseudomonas stutzeri* A1501. Acta Microbiol Sin.

[22] Carbon S, Ireland A, Mungall CJ, Shu S, Marshall B, Lewis S, AmiGO Hub, Web Presence Working Group (2009). AmiGO: online access to ontology and annotation data. Bioinformatics.

[23] Wass MN, Sternberg JE (2008). ConFunc - functional annotation in the twilight zone. Bioinformatics.

[24] Conesa A, Gotz S (2008). Blast2GO: a comprehensive suite for functional analysis in plant genomics. Int J Plant Genomics.

[25] The InterPro Consortium (2002). InterPro: An integrated documentation resource for protein families, domains and functional sites. Brief Bioinform.

[26] Thomas PD, Campbell MJ, Kejariwal A, Mi H, Karlak B, Daverman R, Diemer K, Muruganujan A, Narechania A (2003). PANTHER: a library of protein families and subfamilies indexed by function. Genome Res.

[27] Finn RD, Bateman A, Clements J, Coggill P, Eberhardt RY, Eddy SR, Heger A, Hethweington K, Holm L, Mistry J, Sonnhammer ELL, Tate J, Punta M (2014). The Pfam protein families database. Nucleic Acids Res.

[28] Sigrist CJA, Cerutti L, De Castro E, Langendijk-Genevaux PS, Bulliard V, Bairoch A, Hulo N (2010). PROSITE, a protein domain database for functional characterization and annotation. Nucleic Acids Res.

[29] Adler J, Parmryd I (2010). Quantifying colocalization by correlation: the pearson correlation coefficient is superior to the mander’s overlap coefficient. Wiley InterScience.

